# Sexual behaviour of people living with HIV attending a tertiary care government hospital in Kathmandu, Nepal: a cross sectional study

**DOI:** 10.1186/s13104-015-1559-0

**Published:** 2015-11-02

**Authors:** Mirak Raj Angdembe, Shyam Prasad Lohani, Deepak Kumar Karki, Kreepa Bhattarai, Niraj Shrestha

**Affiliations:** Department of Public Health, Central Institute of Science and Technology, Pokhara University, Kathmandu, Nepal; Centre for Health Research and International Relations, Nobel College, Pokhara University, Kathmandu, Nepal; National Centre for AIDS and STD Control, Kathmandu, Nepal; Braun School of Public Health and Community Medicine, Hebrew University, Jerusalem, Israel; Melbourne School of Population and Global Health, The University of Melbourne, Melbourne, Australia

**Keywords:** Antiretroviral therapy, Sexual behaviour, HIV, AIDS, Nepal

## Abstract

**Background:**

Clinical improvements that follow antiretroviral therapy (ART) may lead to increase or resumption of high risk activities that could unintentionally result in HIV transmission. The objective was to investigate whether treatment status is a significant predictor of sexual risk behaviour (unprotected sex).

**Methods:**

A cross sectional study was conducted among 160 people living with HIV (PLHIV) (89 ART experienced and 71 ART naïve) attending Sukraraj Tropical and Infectious Disease Hospital in Kathmandu, Nepal. A structured questionnaire was used for data collection. Logistic regression with stepwise modeling was used to obtain adjusted odds ratios (OR) with 95 % CI.

**Results:**

In this study, 92 % of sexually active respondents reported sex with a regular partner. ART experienced PLHIV were significantly more likely to report consistent condom use with their regular partners compared to ART naïve PLHIV (83 vs. 53 %; *P* = 0.006) during the past six months. In multivariate analysis, sex (OR = 4.59, 95 % CI: 1.15–18.39), treatment status (OR = 4.76, 95 % CI: 1.29–17.52) and alcohol consumption during last sex with regular partners (OR = 14.75, 95 % CI: 2.75–79.29) were significantly associated with unprotected sex.

**Conclusion:**

ART naïve PLHIV were five times more likely to exhibit sexual risk behaviour (have unprotected sex) than ART experienced PLHIV. Thus the study provided no evidence to suggest that ART experienced PLHIV exhibit greater sexual risk behaviour compared to ART naïve PLHIV. Prevention programmes need to emphasize on counselling to PLHIV and their regular partners with focused interventions such as couple counselling and education programmes.

**Electronic supplementary material:**

The online version of this article (doi:10.1186/s13104-015-1559-0) contains supplementary material, which is available to authorized users.

## Background

The development of antiretroviral therapy (ART) has led to reduction in mortality and morbidity rates among people living with HIV (PLHIV) [1, 2]. An early initiation of ART will lead to near normal quality of life and lifespan [3–5]. ART reduces the concentration of the virus in plasma which is a primary determinant of the risk of transmission [6]. Reduction in risk of transmission to sexual partners among those initiating ART will depend upon viral load in plasma and genital tract [1, 7]. Even with the infected partner on effective ART and undetectable viral loads, transmission of HIV although rare has been documented [8–10]. Thus, risk is substantially reduced [11–15] but not completely eliminated.

Clinical improvements that follow ART may lead to increase or resumption of high risk activities that could unintentionally result in HIV transmission [16]. Optimism related to the success of ART in slowing disease progression, reducing viral load, and improving health status [17] might be crucial factors determining sexual risk behaviours. Since highly active ART (HAART) became available, there has been an increase in sexual risk behaviour among the PLHIV [18–22]. Beliefs about reduced risk for HIV transmission with ART may also influence the behaviour [23]. In contrast, a meta-analysis did not find an increase in sexual risk behaviour following ART. However, it did report a higher likelihood of unprotected sex in individuals who believed that therapy prevented transmission or who were less concerned about transmission given the availability of ART [24]. Additionally, use of ART was not associated with increased sexual risk behaviour in PLHIV undergoing ART [25, 26]; homosexual men receiving ART [27]; HIV infected injecting drug users [16]. Some studies suggest a decrease or lower sexual risk behaviour following ART [22, 28–30].

With increased sexual risk behaviour there is also the possibility of transmission of drug resistant HIV strains. Between 7 and 17 % of newly infected people in high income countries carry at least one major drug resistance mutation [31, 32]. On the other hand, middle and low income countries are estimated to have a lower prevalence of transmitted drug resistance at around 7 % [32]. This situation however is likely to change with increased availability of ART. Evidence from East and Southern Africa already show a rapid increase in transmitted drug resistance following ART rollout [33]. In addition to drug resistance, there is also the risk of superinfection, which is defined as an infection with a second strain following the initial infection and immune response [34]. With an estimated incidence of 0–7.7 % per year, superinfections have adverse effects on clinical outcomes and might affect ART scale up plans [35]. Thus even with the possibility of ART for prevention, the importance of safe sexual practice cannot be undermined.

In Nepal, ART was initiated in 2004 by Sukraraj Tropical and Infectious Disease Hospital, a tertiary care hospital run by Government of Nepal in Teku, Kathmandu [36]. By the end of 2014, the government was providing ART free of cost to 10,407 PLHIV from 53 ART centres across the country [36]. According to national guidelines, ART is initiated when the CD4 cell count is ≤500 cells/mm^3^ with priority to those with WHO clinical stage 3 or 4 disease. It is also recommended to initiate ART in people with active tuberculosis (TB) disease and hepatitis B virus co-infection with severe liver disease, all pregnant and breastfeeding women with HIV, and all individuals with HIV in serodiscordant relationships, regardless of CD4 cell count [37]. Those who do not fulfil these criteria are considered Pre ART (ART naïve) individuals who are regularly monitored for CD4 cell count and treated for opportunistic infections (OI) when necessary.

With increase in treatment coverage, greater number of people are expected to survive in Nepal, indicating an increase in prevalence of HIV infection among treated populations. In Nepal, it is estimated that more than 85 % of the total new HIV infections are the result of sexual transmission [38]. A small change in sexual risk behaviour in such population could have a large impact on the course of the epidemic. Studies from South Africa and Uganda have developed models which suggest that increases in sexual risk behaviour by those initiating ART could reduce expected declines in HIV incidence [39, 40]. It has been estimated that the probability for HIV epidemic eradication through widespread ART use is 85 % if sexual risk behaviour decreases [41]. If levels of risky sex remain stable, that probability decreases to 50 %, and decreases further in the presence of increased sexual risk behaviours [41].

Thus this study was conducted to learn more about the impact of ART on sexual risk behaviour. The objective was to investigate whether treatment status (ART experienced vs. ART naïve) is a significant predictor of sexual risk behaviour among PLHIV attending a tertiary care government hospital in Kathmandu, Nepal.

## Methods

This was a cross sectional study where sexual behaviour of PLHIV was investigated. We collected data from the ART clinic of Sukraraj Tropical and Infectious Disease Hospital, Teku, Kathmandu, one of the tertiary care hospitals in the capital city of Nepal, during July–August 2010. This is the largest ART centre in the country where PLHIV are referred from various districts.

A total of 160 PLHIV (89 ART experienced and 71 ART naïve) were conveniently enrolled for structured interviews. It was assumed that the PLHIV visit the center in a natural random order. Every PLHIV who visited the center during clinic hours was considered for the study with enrollment based on certain criteria. The study included only those PLHIV who were enrolled in HIV care for at least six months since HIV was diagnosed, who were 18 years of age or older and had not initiated ART (ART naïve PLHIV) or ART experienced for at least six months. PLHIV who were hearing and speech impaired, seriously ill or admitted to hospital and those who could not give informed consent were not enrolled in the study. It was presumed that pregnancy and TB infection would affect the sexual behaviour of PLHIV. Thus they were also excluded.

PLHIV’s medical records maintained in the ART clinic were reviewed to record the latest CD4 cell count in the past six months and date when HIV was diagnosed. Measure of CD4 cell count could not be obtained for all respondents because medical records were not updated. Names and other identifying information were not obtained. Interviews were conducted using a structured questionnaire to collect behavioral information. The questionnaire (Additional file 1) was adapted from standardized questions used in integrated biological and behavioral surveillance (IBBS) surveys in Nepal with established validity and reliability [42]. It was then discussed among experts for further validation. The questionnaire was pre-tested among 10 % (16) of the anticipated sample size at the study site itself. During pre-testing, respondents hesitated to answer the question on whether they had penile-anal sex with men or women. The concept was detested and considered unnatural. Thus the definition of sex was limited to penile vaginal insertive sexual events and the study inquired only heterosexual behavior.

The key themes of analysis for sexual risk behaviour were type of partners (regular, commercial and casual partners), knowledge of partner’s HIV status, disclosure of own HIV status to partners, alcohol consumption during last sex with partners, condom use during last sex and consistent condom use with partners. The recall reference period was six months. Sex was defined as penile vaginal insertive sexual events during the past six months. A person reporting to be involved in sex with one or more sexual partners during the past six months was defined as sexually active. A commercial partner was defined as a partner to whom the respondent paid in cash or kind in exchange for sex. A regular partner was defined as a spouse or someone with whom the respondent had a stable relationship such as boyfriend/girlfriend or live-in sexual partner who was never paid for sex. A casual partner was defined as a sexual partner with whom the respondent had sex only once or rarely, was not living with or married to and never paid (in cash or kind) for sex. Unprotected sex was defined as no condom use at last sex or inconsistent (sometimes or never) condom use during sex with regular partners during the past six months.

The process of data management started at the study site with review of the completed questionnaire for possible errors. Data were entered in SPSS (ver.17) and analyzed using SPSS (ver.17) and Stata (ver.12). Before analysis, the data were checked and rechecked for missing values by analyzing frequencies. Fisher’s exact test was used to determine statistically significant differences for categorical variables. Logistic regression analysis was performed to examine the effects of independent variables on sexual behaviour with regular partners. The outcome variable sexual behaviour was coded as *0* = *protected sex* and *1* = *unprotected sex*. Initially, independent variables were included in the model one at a time to examine their univariate relationship with sexual behaviour. This was followed by the development of a multivariate model. Using an empty model to begin with, variables were fitted one at a time (stepwise modeling). At each stage, the least significant variable was excluded until the model contained only statistically significant factors. A *P*-value of <0.05 was considered to be statistically significant.

The study protocol was reviewed and approved by the Ethical Review Board of the Faculty of Health Sciences, Nobel College, Pokhara University and Sukraraj Tropical and Infectious Disease Hospital, Teku. The respondents were given complete and clear information about the study being conducted enabling them to make an informed decision whether or not to participate in the study. The informed consent was obtained verbally from all respondents.

## Results

The mean age of the respondents was 34.5 years (SD = ±6.62). Other background characteristics of respondents are presented in Table 1. Majority of the respondents were Hindus (70 %), married or living with a partner (63 %) currently employed (80 %), having secondary level education (42 %) and earning monthly income in the range of Nepalese rupees (NRs.) 5000–10,000 (53 %) (1US Dollar = NRs. 105, as of September 2015 [43]). Among those employed, most were engaged in agriculture (23 %) followed by business (19 %) (not shown in table). The mean time since HIV diagnosed was 41 months (SD = ±3.2) and the mean CD4 cell count in the past six months (n = 146) was 242 cells/mm^3^ (SD = ±13.1) (Not shown in table).

**Table 1:** Socio demographic characteristics of the respondents

Variables (N = 160)	n (%)
Sex, female	58 (36.3)
Current residence, inside Kathmandu valley	81 (50.6)
Religion	
Hindu	107 (66.9)
Buddhist	21 (13.1)
Christian	26 (16.2)
Islam	6 (3.8)
Ethnicity^a^	
Brahmin/Chhetri	73 (45.6)
Janajati	68 (42.5)
Others	19 (11.9)
Marital status	
Married or living with a partner	101 (63.1)
Unmarried/single	20 (12.5)
Divorced/separated	12 (7.5)
Widow/widower	27 (16.9)
Education status	
No education	48 (30.0)
Primary (1–5)	26 (16.2)
Secondary (6–10)	67 (41.9)
Higher	19 (11.9)
Unemployed	32 (20.0)
Monthly income in Nepali rupees^b^ (n = 98)	
<5000	28 (28.6)
5000–10,000	52 (53.1)
>10000	18 (18.3)

^a^ *Janajati* are considered as indigenous population groups who are specifically resided in local areas in various parts of country; whereas *Brahmins/Chhetries* are considered as upper class ethnic groups and have relatively more access to resources, power and identity [54]

^b^1US$ = 105 Nepali rupees

Fifty-two percent (83/160) of respondents reported having had sexual intercourse during the past six months (ART experienced = 51 % and ART naïve = 53 %). Ninety-two percent (76/83) of sexually active respondents reported sex with a regular partner (ART experienced = 93 % and ART naïve = 89 %) (Table 2). A greater number of ART experienced PLHIV reported consistent condom use with their regular partner compared to ART naïve PLHIV (83 vs. 53 %; *P* = 0.006). ART experienced PLHIV reported greater condom use at last sex with their regular partner than ART naïve PLHIV (84 vs. 66 %) but the difference was not statistically significant. Nearly 83 % of ART experienced and 71 % of ART naïve PLHIV knew their regular partner’s HIV status. Likewise, 88 % of ART experienced and 85 % of ART naïve PLHIV disclosed their own HIV status to their regular partner. A majority of the ART experienced (83 %) and ART naïve (82 %) PLHIV did not consume alcohol during last sex with regular partner. Compared to ART experienced PLHIV a greater number of ART naïve PLHIV believed that ART can prevent the transmission of HIV (44 vs. 61 %; *P* = 0.040).

**Table 2:** Sexual behaviour in the previous six months and belief towards ART

Variables	ART experienced n (%)	ART Naïve n (%)	*P* ^*a*^-value
Sexually active in the past six months			
Yes (n = 83)	45 (50.6)	38 (53.5)	0.752
No	44 (49.4)	33 (46.5)	
Sex with (n = 83)			
Regular partner	42 (93.3)	34 (89.5)	0.697
Commercial and casual partners	3 (6.7)	4 (10.5)	
Condom use with regular partners			
Consistent (always)	35 (83.3)	18 (52.9)	0.006
Inconsistent (sometimes and never)	7 (16.7)	16 (47.1)	
Condom use at last sex with regular partner			
Yes	38 (84.4)	25 (65.8)	0.071
No	7 (15.6)	13 (34.2)	
Knowledge of regular partner’s HIV status			
Known	35 (83.3)	24 (70.6)	0.269
Not known	7 (16.7)	10 (29.4)	
Disclosure of HIV status to regular partner			
Yes	37 (88.1)	29 (85.3)	0.745
No	5 (11.9)	5 (14.7)	
Alcohol consumption during last sex with regular partner			
Yes	7 (16.7)	6 (17.6)	1.000
No	35 (83.3)	28 (82.4)	
Can ART prevent the transmission of HIV			
Yes/don’t know	39 (43.8)	43 (60.6)	0.040
No	50 (56.2)	28 (39.4)	
With availability of ART would it make a difference if HIV transmits from you to others			
Yes	78 (87.6)	56 (78.9)	0.195
No/don’t know	11 (12.4)	15 (21.1)	

^a^Fisher’s exact test

Regarding the decision to use/not use condom during last sex with regular partners, a majority (61 %) of the males reported that they themselves took the decision to use/not use condom. However in the case of females, a majority (80 %) reported that their male partner or both took the decision to use/not use condom and only 20 % of them reported that they themselves took the decision (not shown in table). This difference was significant (*P* = 0.004).

In univariate analysis, two variables were significantly associated with unprotected sex (Table 3). However in the final logistic regression model, three variables were significantly associated with unprotected sex: sex (OR = 4.59, 95 % CI: 1.15–18.39), treatment status (OR = 4.76, 95 % CI: 1.29–17.52) and alcohol consumption during last sex with regular partners (OR = 14.75, 95 % CI: 2.75–79.29). Sex was not significant in univariate analyses but was found to be significant in multivariate analysis.

**Table 3:** Predictors of unprotected sex with regular partners

Variables	Unprotected n (%)	Protected n (%)	Univariate OR (95 % CI)	*P*-value	Multivariate OR (95 % CI)	*P*-value
Sex						
Female	9 (45.0)	11 (55.0)	2.46 (0.84–7.15)	0.100	4.59 (1.15–18.39)	0.031
Male	14 (25.0)	42 (75.0)				
Marital status						
Not living with a partner	1 (50.0)	1 (50.0)	2.36 (0.14–39.5)	0.549		
Living with a partner	22 (30.0)	52 (70.0)				
Education level^b^						
Formal education	18 (30.5)	41 (69.5)	1.05 (0.32–3.43)	0.931		
No formal education	5 (29.4)	12 (70.6)				
Occupation						
Unemployed	6 (46.2)	7 (53.8)	2.32 (0.68–7.89)	0.178		
Employed	17 (26.9)	46 (73.1)				
Treatment status						
ART naïve	16 (47.1)	18 (52.9)	4.44 (1.55–12.76)	0.006	4.76 (1.29–17.52)	0.019
ART experienced	7 (16.7)	35 (83.3)				
Partner’s HIV status						
Not known	7 (41.2)	10 (58.8)	1.35 (0.43–4.27)	0.606		
HIV negative	1 (6.7)	14 (93.3)	0.14 (0.02–1.15)	0.068		
HIV positive	15 (34.1)	29 (65.9)				
Disclosure of own HIV status						
No	5 (50.0)	5 (50.0)	2.67 (0.69–10.31)	0.155		
Yes	18 (27.3)	48 (72.7)				
Alcohol consumption during last sex						
Yes	9 (69.2)	4 (30.8)	7.88 (2.11–29.45)	0.002	14.75 (2.75–79.29)	0.002
No	14 (22.2)	49 (77.8)				
Time since HIV was diagnosed^a^						
≤24 months	14 (40.0)	21 (60.0)	2.37 (0.87–6.46)	0.091		
>24 months	9 (21.9)	32 (78.1)				
CD4 Cell count (cells/mm^3^)^a^						
≤224	12 (37.5)	20 (62.5)	2.18 (0.75–6.28)	0.151		
>224	8 (21.6)	29 (78.4)				
Can ART prevent the transmission of HIV						
Yes/don’t know	14 (35.0)	26 (65.0)	1.61 (0.60–4.37)	0.345		
No	9 (25.0)	27 (75.0)				
With availability of ART would it make a difference if HIV transmits from you to others						
Yes	18 (27.7)	47 (72.3)	0.46 (0.12–1.70)	0.243		
No/don’t know	5 (45.4)	6 (54.6)				

^a^Dichotomization based on sample median

^b^Formal education includes primary (grade 1–5), secondary (grade 6–10), higher secondary/high school (grade 11–12), under graduate and post graduate

## Discussion

Rapid development in the field of ART has led to reduction in morbidity and mortality among PLHIV. However, there is a growing concern over sexual risk behaviour of PLHIV on ART. The changes in sexual risk behaviour of PLHIV with the initiation of ART might have important implications on the way counseling may be designed and implemented. An increase in sexual risk behaviour with therapy will be a matter of great concern from a prevention of transmission perspective. If not intervened, it may further fuel the epidemic.

In this study, only around half of the respondents reported to be sexually active in the past six months. It is possible that lower sexual activity may be a result of symptomatic disease or a result of adverse effects of ART [23]. The psychological trauma resulting from a positive diagnosis might also reduce sexual desire. A majority of the respondents in both groups (ART experienced and ART naïve) had sex only with their regular partners in the past six months. Those who had other types of partners always used condoms during sex except for one male ART naïve PLHIV who never used condom with his casual partner. This meant that sexual risk behaviour was mostly dependent upon the respondents’ condom use with their regular partners. This is similar to other studies where most reported sex was with regular partners [17, 30, 44]. In India, 96 % of those who were sexually active in the past six months reported sex with a regular partner [17]. However, we should consider the influence of both recall and social desirability bias on responses related to sexual behaviour when interpreting these results.

In regular partner relationship, non-disclosure of self’s HIV status, lack of knowledge of partner’s HIV status in combination with unprotected sex lays out favourable circumstances for transmission of HIV to serodiscordant partners. Furthermore unprotected sex with regular partner also carries the risk of unwanted pregnancy and the transmission of HIV virus to the child. Nearly a fifth (20 %) of the respondents who had sex with regular partners were in a discordant relationship with an HIV negative partner but a majority of them (93 %) had protected sex. Likewise, 58 % were in a concordant relationship with an HIV positive partner but a relatively lower percentage (66 %) reported protected sex. Unprotected sex with HIV positive partners carries the risk of becoming infected with resistant viral strains or reinfection with novel strains [30].

With regular partners, ART experienced PLHIV were more likely to have protected sex compared to ART naïve PLHIV, which was similar to other studies conducted in a developing country setting [17, 25, 30]. Thus, the study provides no evidence to suggest that sexual risk behaviour may actually increase with initiation of ART in this context [23, 25, 26, 28–30]. Belief about the reduced severity and threat of the disease due to the availability of HAART as well as those about HAART related HIV transmission might have contributed to increased sexual risk behaviour [24] particularly among ART naïve PLHIV compared to ART experienced PLHIV. On the other hand, lower risk behaviour among ART experienced may be explained when taking into account their larger mean time since HIV diagnosis (43 months) compared to ART naïve PLHIV (29 months). This means a longer time had elapsed since getting tested and diagnosed with greater chances of having advanced HIV disease as well. Consequently they may perceive the seriousness of their illness differently and should have had several contacts with health workers including counsellors who conveyed prevention and protection messages during the course of treatment. This may have contributed to lower sexual risk behaviour. In addition, similar to a study in India, the belief that condoms are needed for ART to be effective might have facilitated consistent condom use with regular partners [45] among ART experienced PLHIV. Among married couples, the desire to have children might also be a predictor of unprotected sex with regular partners [46] but it was not investigated in this study. In order to get a clear understanding of the underlying reasons for unprotected sex, it might be necessary to investigate individual motives through a qualitative inquiry.

Females were more likely to have unprotected sex compared to males. The decision to use/not use condom was taken mostly by the males. Females seem to have very little say when it comes to deciding on condom use. This might have contributed to this finding. A study from Asia found that wealthier and highly educated married women were more likely to report that they can refuse sexual intercourse and ask their husbands to use a condom [47]. Additionally the study had also found that women’s overall involvement in making family decisions empowers them to negotiate safer sex [47].

Alcohol consumption during last sex with regular partners was one of the significant predictors of unprotected sex. The use of alcohol may decrease the ability to make rational judgements and provoke risk behaviours as unprotected sex. Studies conducted in different populations and high risk sub-groups have attempted to examine the relationship where a number of them report an association between risk behaviour and alcohol consumption [45, 46, 48–52]. On the contrary, a meta-analysis to assess the relationship of alcohol use and condom use did not find an association [53].

Similar to the limitations of other previous studies, [44] this current cross-sectional study could not derive causal inferences. Further, the study defined sex as penile vaginal insertive sexual events which meant that other risky sexual behaviour including penile-anal sex between men and between men and women were not accounted for. In the multivariate analysis, the reduction in sample size may have resulted in wider confidence intervals. Thus, the conclusions may be tentative, especially with regards to the non-significant findings. The fact that sex of the person was statistically significant only in the multivariate analysis, once ART status was controlled for may indicate an interaction effect, but this could not be tested with the small sample size. These limitations should be taken into consideration when interpreting the results of this study. Also, since sexual behaviour was self-reported it may have been underestimated [28, 30, 44]. Furthermore, it is also important to note the possible influence of social desirability on study results.

## Conclusions

Treatment status was found to be a significant predictor of sexual risk behaviour with regular partners. ART naïve PLHIV were five times more likely to exhibit sexual risk behaviour (have unprotected sex) with regular partners than ART experienced PLHIV. Regular partners such as spouses are an important group as far as prevention of transmission is concerned as they are the most likely partners.

In addition to having targeted prevention programmes for high risk sub-populations, the study highlights the need to emphasize on counselling to PLHIV and their regular partners. Focused interventions such as couple counselling and education programmes are required to be built into the total HIV care and treatment programme. Practicing safer sex with regular sexual partners and refraining from alcohol use during sex should be the critical messages. Additionally, programmes should focus on empowering women to make decisions regarding condom use during sex. In the future, a longitudinal study with adequate sample size can provide causal inferences regarding the impact of ART on sexual behaviours.

## Additional file

10.1186/s13104-015-1559-0 English version of the questionnaire.
