# Supplementary material for: Sexual behaviour of people living with HIV attending a tertiary care government hospital in Kathmandu, Nepal: a cross sectional study
Source: BMC Res Notes. 2015 Nov 2;8:629. doi: 10.1186/s13104-015-1559-0 (PMC4630840; doi:10.1186/s13104-015-1559-0)
Supplement: Supplementary file 1 — 10.1186/s13104-015-1559-0 English version of the questionnaire. [file 13104_2015_1559_MOESM1_ESM.docx]

**QUESTIONNAIRE**

| **IDENTIFICATION NUMBER:** |  |
| --- | --- |

____________________________________________________________________________

Did the respondent abandon the interview?

1. Yes (Specify the number of the last question completed: Q ____)

2. No

(If the respondent abandoned the interview, state the reason­­­­­­­­­­­­­­­­­­__________________________)

Interviewer Name: ____________________ Signature: _____________________

Date of Interview: _____ / _____ / 2010

Checked by the supervisor: Signature: _________________ Date: ____/ ____ / 2010

**____________________________________________________________________________**

**SCREENING QUESTIONS**

Do not take interview if respondent is hearing or speech impaired, pregnant, seriously ill or admitted to hospital, who cannot give informed consent and who is suffering from TB/HIV co-infection

*[Cross check all answers with patient medical record]*

1. 18 years or older (If **Yes** go to 2, else **STOP**)
2. Enrolled in HIV care for at least 6 months since HIV was diagnosed (If **Yes** go to 3, else **STOP**)
3. Taking ART (If **Yes** go to 4, else go to 5)
4. Taking ART for at least 6 months (If **Yes** go to 5, else **STOP**)
5. **START** Interview

**1. SOCIO-DEMOGRAPHIC INFORMATION**

| **Q. N.** | **Questions and Filters** | **Coding Categories** | **Skip** |
| --- | --- | --- | --- |
| 101. | Sex | *Male…………………………..1*  *Female……………………….2* |  |
| 102. | How old are you? | *Age ………………….*  ***(write the completed years)*** |  |
| 103. | Where do you live now?  ***(Name of current place of residence)*** | *District:­­­­­­­­­­­­­­­­­­________________*  *VDC/Municipality:_____________* |  |
| 104. | What is your religion?  ***(Specify the religion)*** | *Religion__________________*  *(Specify)*  *Code No……..…………* |  |
| 105. | What is your caste?  ***(Specify Ethnic Group/Caste)*** | *Ethnicity/Caste______________*  *(Specify)*  *Code No…………….….* |  |
| 106. | What is your educational status?  ***(Circle '0' if illiterate, '19' for the literate***  ***without attending the school, and write***  ***exact number of the passed grade)*** | *Illiterate .................................0*  *Literate................................19*  *Grade..............................*  *(****write the completed grade****)*  *If more than high school, specify the degree completed ___________________________* |  |
| 107. | What is your present marital status? | *Married or living with a partner………………………… 1*  *Divorced/Separated…..............2*  *Widow/Widower.......................3*  *Unmarried/Single.....................4* | 107.1  107.2 |
| 107.1 | Are you living with your husband/wife? | *Yes……………………………..1*  *No………………………………2* | 108 |
| 107.2 | Are you living with any other sexual partner? | *Yes……………………………..1*  *No………………………………2*  *No response ......................... 99* | 108 |
| 108. | What is your current occupation? | *Occupation________________*  *(Specify)*  *House wife…………………1*  *Unemployed……………….2* | 201 |
| 109. | What is your average weekly income from the above-mentioned source? | **___________________**NRs. |  |

**2. INFORMATION ON SEXUAL BEHAVIOR**

| **Q. N.** | **Questions and Filters** | **Coding Categories** | **Skip** |
| --- | --- | --- | --- |
| 201. | Have you had sexual intercourse in the last 6 months? | *Yes............................................ 1*  *No ............................................. 2*  *No response............................ 99* |  |
| 202. | Did you have sex with a “regular partner” in the last 6 months?  **(Spouse, boyfriend/girlfriend or live-in sexual partners who was never paid for sex)** | *Yes ............................................ 1*  *No.............................................. 2* | 203 |
| 202.1 | The last time you had sex with your current regular partner; did you and your partner use a condom? | *Yes ............................................ 1*  *No.............................................. 2*  *Don't know............................... 98*  *No response............................ 99* |  |
| 202.2 | The last time you had sex with your current regular partner who took the decision to use/not use condom? | *You……………………………….1*  *Your sexual partner….…………2*  *Both…………………….………..3*  *Don’t know……………………....98*  *No response………………….…99* |  |
| 202.3 | How often have you used a condom with your current regular partner in the past 6 months? | *Always……….…………………...1*  *Sometimes ................................ 2*  *Never…………........................... 3*  *Don't know................................ 98*  *No response............................. 99* |  |
| 202.4 | Do you know the HIV status of your current regular partner? | *HIV positive………………………1*  *HIV negative……………….…….2*  *Don’t know………………………98*  *No response……………………99* |  |
| 202.5 | Have you disclosed your HIV status to your current regular partner? | *Yes ............................................ 1*  *No.............................................. 2*  *No response............................ 99* |  |
| 202.6 | The last time you had sex with your current regular partner; did you consume drinks containing alcohol? | *Yes ............................................ 1*  *No.............................................. 2*  *Don't know.............................. 98*  *No response............................ 99* |  |
| 203. | Did you have sex with a “commercial sex worker” in the last 6 months?  **(someone the respondent paid in cash or kind in exchange for sex)** | *Yes ............................................ 1*  *No.............................................. 2* | 204 |
| 203.1 | The last time you had sex with a commercial partner; did you and your partner use a condom? | *Yes ............................................ 1*  *No.............................................. 2*  *Don't know.............................. 98*  *No response............................ 99* |  |
| 203.2 | The last time you had sex with a commercial partner who took the decision to use/not use condom? | *You……………………………….1*  *Your sexual partner…………….2*  *Both…………………….………..3*  *Don’t know…………………..…..98*  *No response………….…………99* |  |
| 203.3 | How often have you used a condom with commercial partner in the past 6 months? | *Always……………..……………...1*  *Sometimes ................................. 2*  *Never………............................... 3*  *Don't know................................ 98*  *No response............................. 99* |  |
| 203.4 | Do you know the HIV status of your last commercial partner? | *HIV positive………………………1*  *HIV negative……….…………….2*  *Don’t know………………………98*  *No response……………………99* |  |
| 203.5 | Did you disclose your HIV status to your last commercial partner? | *Yes ............................................ 1*  *No.............................................. 2*  *No response............................ 99* |  |
| 203.6 | The last time you had sex with a commercial partner; did you consume drinks containing alcohol? | *Yes ............................................ 1*  *No.............................................. 2*  *Don't know.............................. 98*  *No response............................ 99* |  |
| 204. | Did you have sex with a “casual partner” in the last 6 months?  **(someone with whom the respondent had sex only once or rarely, was not living with or married to and never paid (in cash or kind)) for sex)** | *Yes ............................................ 1*  *No.............................................. 2* |  |
| 204.1 | The last time you had sex with a casual partner; did you and your partner use a condom? | *Yes ............................................ 1*  *No.............................................. 2*  *Don't know.............................. 98*  *No response............................ 99* |  |
| 204.2 | The last time you had sex with a casual partner who took the decision to use/not use condom? | *You……………………………….1*  *Your sexual partner………………2*  *Both………………………………..3*  *Don’t know……………………….98*  *No response…………………….99* |  |
| 204.3 | How often have you used a condom with casual partners in the past 6 months? | *Always………………….………...1*  *Sometimes ................................. 2*  *Never………............................... 3*  *Don't know................................ 98*  *No response............................. 99* |  |
| 204.4 | Do you know the HIV status of your last casual partner? | *HIV positive………………………1*  *HIV negative…………………….2*  *Don’t know………………………98*  *No response……………………99* |  |
| 204.5 | Did you disclose your HIV status to your last casual partner? | *Yes ............................................ 1*  *No.............................................. 2*  *No response............................ 99* |  |
| 204.6 | The last time you had sex with a casual partner; did you consume drinks containing alcohol? | *Yes ............................................ 1*  *No.............................................. 2*  *Don't know.............................. 98*  *No response............................ 99* |  |

**3. Belief towards ART**

| **Q. N.** | **Questions and Filters** | **Coding Categories** | **Skip** |
| --- | --- | --- | --- |
| 301. | Do you think ART can prevent the transmission of HIV? | *Yes ....................................... 1*  *No......................................... 2*  *Don’t know…....................... 98* |  |
| 302. | With availability of ART would it make a difference if HIV transmits from you to others? | *Yes ....................................... 1*  *No......................................... 2*  *Don’t know…....................... 98* |  |
